# Systemic delivery of menstrual blood stem cells is more effective in preventing remote organ injuries following myocardial infarction in comparison with bone marrow stem cells in rat

**DOI:** 10.22038/IJBMS.2023.67574.14809

**Published:** 2023

**Authors:** Mahmood Manshori, Somaieh Kazemnejad, Nasim Naderi, Maryam Darzi, Nahid Aboutaleb, Hannaneh Golshahi

**Affiliations:** 1 Nanobiotechnology Research Center, Avicenna Research Institute, ACECR, Tehran, Iran; 2 Rajaie Cardiovascular Medical and Research Center, Iran University of Medical Sciences, Tehran, Iran; 3 Physiology Research Center, Iran University of Medical Sciences, Tehran, Iran; 4 Department of Physiology, Faculty of Medicine, Iran University of Medical Sciences, Tehran, Iran

**Keywords:** Bone marrow stem cell, Kidney, Liver, Menstrual blood stem cells, Myocardial infarction, Remote organ injury

## Abstract

**Objective(s):**

Remote organ injury is a phenomenon that could happen following myocardial infarction (MI). We evaluated the potency of menstrual blood stromal (stem) cells (MenSCs) and bone marrow stem cells (BMSCs) to alleviate remote organ injuries following MI in rats.

**Materials and Methods:**

2 × 10^6^ MenSCs or BMSCs were administrated seven days after MI induction via the tail vein. Four weeks after cell therapy, activities of aspartate aminotransferase (AST), urea, creatinine, and Blood Urea Nitrogen (BUN) were evaluated. The level of tumor necrosis factor-α (TNF-α), interleukin (IL)-1β, and IL-6 were determined by ELISA assay. The expression of Nuclear Factor-κB (NF-κB) was evaluated by immunohistochemical staining. Apoptosis activity and tissue damage were also determined by TUNEL and H&E staining, respectively.

**Results:**

MenSCs and BMSCs administration caused a significant reduction in AST, urea, and BUN levels compared with the MI group. In addition, systemic injection of MenSCs significantly decreased the IL-1β level compared with BMSCs and MI groups (*P*<0.05 and *P*<0.01 respectively). Apoptosis in injured kidneys was noticeably diminished in MenSCs-treated rats compared with BMSCs administrated and MI groups (*P*<0.05 and *P*<0.05, respectively). In hepatic tissue, limited numbers of TUNEL-positive cells were detected in all groups. Interestingly, MenSCs therapy evoked inhibition of NF-κB in the kidney strikingly. Although, no significant NF-κB expression was observed in hepatic tissue in any group (*P*>0.05).

**Conclusion:**

MenSCs are probably more protective than BMSCs on remote organ injuries following MI via decreasing cell death and immunoregulatory properties.

## Introduction

Myocardial infarction (MI) is considered a major clinical problem and a main cause of death and disability worldwide (1). Heart failure (HF) is a common complication of MI that could result in late morbidity and mortality in patients. HF can cause, not only cardiac disorders, but also dysfunction in the other vital organs (e.g., kidney, liver, and lung) (2). The heart, liver, and kidney are in close relation to each other, so impairment of cardiac function may lead to hepatic and renal dysfunction and vice versa (3). Hypoperfusion-induced hypoxia altered microcirculation, oxygenation, functional derangement, and tissue injuries (4). So, damage to these vital organs is responsible for high mortality and morbidity in patients with MI (5). On the other hand, MI can affect the function of the kidney and liver by activating inflammatory cells and overexpression of pro-inflammatory cytokines such as tumor necrosis factor-α (TNF-α), interleukin (IL)-1β, and IL-6 (6). In addition, cardiomyocytes in response to the hypoxic condition and inflammatory microenvironment, produce an abundance of reactive oxygen species (ROS) (7). Numerous pieces of evidence support the role of ROS in the promotion of cell death through necrosis and/or apoptosis (8). MI can lead to developing hepatic disorders including reduced hepatic blood flow, increased hepatic vein pressures, decreased arterial saturation, intracellular calcium overload, hepatocellular necrosis, and fibrosis (9). Also, vascular endothelial cell damage, formation of granulation tissue, reduced glomerular function, and tubular injury are the most important outcomes in renal tissue following MI (10).

In recent years, bone marrow-derived stem cells (BMSCs) are the most frequently used stem cells in cell-based therapy (11). Evidence has also shown that the potential of BMSCs in the regenerative medicine field is based on their multilineage differentiation potency, immunomodulatory and self-renewal capacities, rapid proliferation, low immunogenicity, and high portability (12). 

Nevertheless, BMSCs show distinct disadvantages such as limited availability, invasive and painful sample collection, and limited proliferation capacity (13). Instead, human menstrual blood-derived stromal stem cells (MenSCs) have been introduced as a population of mononuclear cells from menstrual blood that have unique features including multipotency, continuous source, procurement via a non-invasive procedure, lack of ethical issues, low immunogenicity, self-renewal ability, and higher growth ability compared with the other types of MSCs (14-16). In addition, the ability of these cells to differentiate into different cell lineages such as respiratory epithelial, neurocytic, cardiomyocytic, endothelial, pancreatic, hepatic, adipocytic, and osteogenic was demonstrated by the expression of specific proteins (16-18). 

MenSCs modulate immune responses by inhibiting the proliferation of various immune cells including T lymphocytes, B lymphocytes, dendritic cells, and natural killer cells (19). They can also promote regulatory T cells by cell contact-dependent mechanisms (20). Researchers have proved that MenSCs inhibited cell death by secreting paracrine factors, including vascular endothelial growth factor (VEGF), hypoxia-inducible factor-1 alpha (HIF-1α), hepatocyte growth factor (HGF), and fibroblast growth factor (FGF) (14, 21). According to our previous studies, we found that the MenSCs-derived conditioned medium (CM) contained various growth factors such as epidermal growth factor (EGF), basic fibroblast growth factor (bFGF), and insulin-like growth factor-1 (IGF-1) (22). In addition, MenSCs have shown multiple positive therapeutic effects for improving damaged myocardium by preventing apoptosis, increasing vascular density, and recruiting endogenous progenitor stem cells (23). It has been shown that the bFGF level in MenSCs-derived CM is higher than BMSC-CM under hypoxic conditions (24).

There have been few studies that investigated the consequences of MI on remote organs and this is the first attempt to compare the therapeutic potential of systemic administration of BMSCs (as the most common stem cell used in stem cell therapy) with MenSCs on hepatic and renal injuries after induction of MI in the preclinical phase. 

## Materials and Methods

***Ethics***

The animal experiment was approved by the Animal Ethical Committee of Iran University of Medical Sciences, Tehran, Iran (IR.IUMS.FMD.REC.1399.542). The study followed the Guide for the Care and Use of Laboratory Animals published by the National Institutes of Health (NIH Publication No. 85-23) (25).

MenSCs and BMSCs were collected from volunteers who agreed and signed the informed consent approved by the medical ethics committee of Avicenna Research Institute (IRCT20180619040147N5). All participants were evaluated by a standard medical history and physical examination. 

***Isolation and culturing of MenSCs and BMSCs***

Isolation of MenSCs was performed as we previously described (25). After collecting, menstrual blood was transferred into the collection tubes containing 2.5 mg/ml fungizone (Gibco, Scotland, UK), 100 μg/ml streptomycin, 100 U/ml penicillin (Sigma-Aldrich, MO, USA), and 0.5 mM EDTA in phosphate-buffered saline (PBS) without Ca^2+^ or Mg^2+^. Menstrual blood mononuclear cells were isolated using density gradient centrifugation in Ficoll-Paque (GE Healthcare, Stockholm, Sweden) and cultured at 37 °C, 5% CO_2 _containing Dulbecco’s Modified Eagle’s Medium/F12 (DMEM-F12) medium (Gibco, UK) supplemented with L-glutamine, non-essential amino acids, penicillin, streptomycin, and 10% fetal bovine serum (FBS). Then, non-adherent cells were washed and the adherent cell population continued until they reached 70–80% confluency. BMSCs were obtained from bone marrow aspirates (5–10 ml) of five healthy donors aged 18–30 years from iliac crests at the Bone Marrow Transplantation Center, Shariati Hospital, Tehran University of Medical Sciences. BMSCs were separated using a combination of density gradient centrifugation and plastic adherence based on the protocol previously studied (16). All experiments were performed with stem cells at the 3–5 passages.

***Characterization of cultured MensSCs and BMSCs by flow cytometry***

Cells (10^5^/100 μl) were incubated separately with phycoerythrin (PE)-conjugated mouse anti-human CD29 (clone 04-MAR; BD Pharmingen), CD45 (clone HI30; BD Pharmingen), and CD105 (clone 43A3; BioLegend). To evaluate OCT-4 expression, the cells were washed with 0.1% saponin as permeabilization and were treated with primary rabbit antihuman OCT-4 antibody (Abcam) followed by incubation with FITC-conjugated goat anti-rabbit Ig (Sigma-Aldrich). Isotype IgG was used as negative control (clone MOPC-21; BD Pharmingen). Finally, all cells were washed with PBS-FBS and fixed in 1% formaldehyde solution. Data were analyzed using a Partec flow cytometer (GmbH, Munster, Germany) (26). 

***Animal modeling protocol***

**Figure 1:**
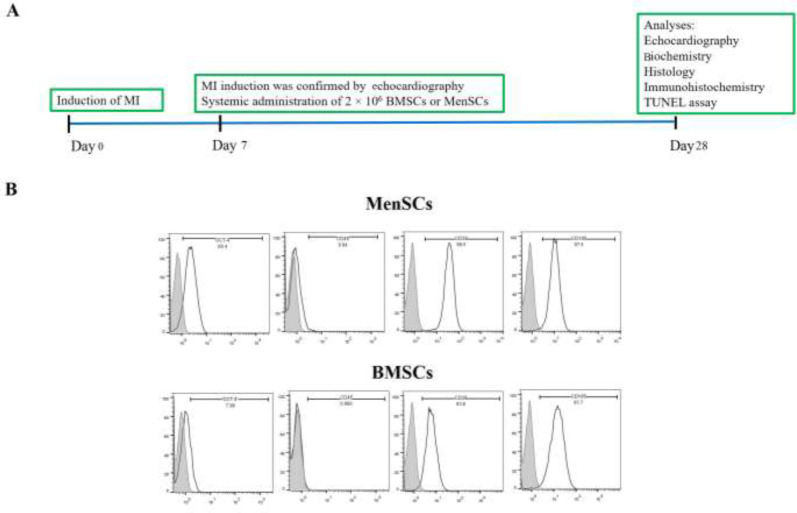
Study design and characteristics of MenSCs and BMSCs. (A) Experimental outline. (B) Evaluating the expression of surface markers of MenSCs and BMSCs by flow cytometry. CD markers are demonstrated as gray curves and the isotype control is shown as white curves

BMSCs: Bone marrow stem cells; MenSCs: Menstrual blood stromal (stem) cells

Animal modeling was done as we previously designated (26). Male Wistar rats weighing 300–350 g were purchased from Iran University of Medical Sciences. The rats were anesthetized with 75 mg/kg ketamine and 5 mg/kg xylazine via intraperitoneal (IP) injection. After intubation and ventilation, the left anterior descending (LAD) artery coronary was permanently ligated with a 6-0 prolene suture. MI induction was confirmed by observation of the pale color development in the distal myocardium after ligation. The sham group underwent the same surgical procedure except for LAD ligature. The chest wall’s muscle layers and skin were closed using absorbable sutures. Post-operation, the animals received cefazolin (25 mg/kg), tramadol (20–30 mg/kg), flunixin (1–2 mg/kg), and warm sterile saline (0.5–1 ml). Seven days after induction of MI, animals were evaluated by echocardiography. Then, animals were randomly divided into the following groups (n=6 per each group): Group 1 (Sham), Group 2 (MI) (200 μl PBS), Group 3 (2 × 10^6^ MenSCs in 200 μl CM of MenSCs), and Group 4 (2 × 10^6^ BMSCs in 200 μl CM of BMSCs). The injections were performed intravenously via the tail vein (Figure 1A).

***Biochemical analysis***

Twenty-eighth days after treatment, blood samples were collected in non-heparinized tubes. Blood samples were centrifuged at 3000 rpm for 10 min, and the obtained serum was stored at −80 °C for further analysis. The serum concentration of aspartate aminotransferase (AST), urea, creatinine, and Blood Urea Nitrogen (BUN) was analyzed with a biochemical auto analyzer (selectra ProM). 

The concentration of IL-1β, IL-6, and TNF-α was also quantified in serum using an enzyme-linked immunosorbent assay (ELISA) kit (R&D Systems) following the manufacturer’s instructions.

***Histological examination***

Thirty-five days after MI induction, samples from the heart, kidney, and liver were collected and fixed with 10% neutral buffered formalin (NBF) and embedded in paraffin. Next, 5 μm tissue sections were deparaffinized and stained with hematoxylin and eosin (H&E) and Masson’s trichrome for histopathological evaluation. 

***Immunohistochemical evaluation***

Briefly, tissue sections were cut into 4-µm thicknesses. After complete deparaffinization and rehydration, samples were treated with 3% H_2_O_2_ in methanol for 10 min to block endogenous peroxidases. In the next step, samples were heated in 0.01M sodium citrate buffer (pH= 6.0) by microwave heating. In order to block, the samples were incubated in 10% goat serum for 40 min at 37 °C. Then, samples were treated with primary antibodies including NF-κB (ab16502, Abcam 1:100) and Anti-Mitochondria, (MAB1273, Merk, 1:150) at 4 °C overnight. In the next step, tissues were treated with the goat polyclonal secondary antibody (HRP) (Biorbyt, UK) for 30 min at room temperature. Positive cells were detected using 100 μl DAB (Sigma, USA). In the final step, tissue sections were counterstained with hematoxylin (Sigma), dehydrated in alcohol, cleared in xylene, and examined by a microscope (Olympus BX51) connected to a digital camera (Olympus DP71). We also quantitatively evaluated the percentage of NF-κB expression by ImageJ software (ImageJ, NIH, Bethesda, MD, USA).

***TUNEL assay***

Apoptotic cell death was detected in kidney and liver tissues using a TUNEL kit (Roche, Mannheim, Germany), according to the manufacturer’s protocol. Briefly, paraffin sections were deparaffinized in xylene and twice washed in PBS. Slices were incubated with the permeabilization solution (0.1% Triton X-100) for 10 min at 4 °C. In the next step, the samples were incubated with 3% H_2_O_2_ for 10 min in a dark place. The sections were incubated in the 50 μl TUNEL reagents for 60 min at 37 °C. Finally, DAB was added for 10 min. The TUNEL-positive cells were counted in five random fields per slide (×400). The apoptotic cell percentage was determined according to the following formula: The number of apoptotic cells/apoptotic cells + non-apoptotic cells) × 100%.

***Statistical analysis***

Results were expressed as mean±SD. Significant differences among groups were determined in three or more groups by one-way ANOVA. *P*-value<0.05 was accepted as statistically significant. All statistical analyses were carried out using SPSS version 20.0, software (IBM Corp, Armonk, NY,USA, http://www.ibm.com). 

## Results

***Characterization of cultured MenSCs and BMSCs***

Immunophenotyping analysis has shown that both MenSCs and BMSCs were positive for CD105 and CD29 and negative for CD45 as a hematopoietic marker. In contrast, OCT-4 as the embryonic marker was only expressed in MenSCs (Figure 1B). 

***MenSCs improved cardiac function and reduced the infarct size***

**Figure 2:**
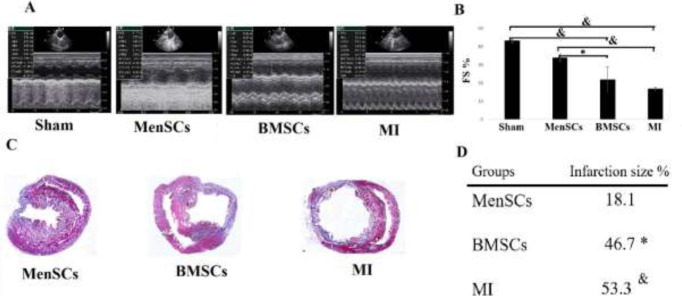
Evaluation of cardiac function and myocardial infarction size (MI size). (A) Representative images of echocardiographic findings in different groups. (B) Echocardiography results revealed a significant increase in FS in rats receiving MenSCs compared with BMSCs and MI groups. (C) Representative heart sections analyzed with Masson's trichrome staining in different groups 35 days after MI induction (D) Quantitative data of the percentage of the LV fibrotic size/LV area among different groups, Scale bar=1.5 mm. *: *P*<0.05, &: *P*<0.01

FS: Fractional shortening; MI: Myocardial infarction; EF: Ejection fraction; BMSCs: Bone marrow stem cells; MenSCs: Menstrual blood stromal (stem) cells

Echocardiographic indexes 35 days post-MI indicated the reduction of fractional shortening (FS) in the MI group compared with the sham group (*P<*0.01). Also, a noticeable improvement in FS was detected in the MenSCs group in comparison with the MI and BMSCs groups (*P<*0.01 and *P<*0.05*,* respectively) (Figure 2A-B). Also, the infarct size was expressed as the ratio of the infarct area (blue) to the entire left ventricular area. MenSCs treatment significantly reduced the infarct size compared with BMSCs and MI group (*P<*0.01 and *P< *0. 05*, *respectively) (Figure 2C-D).

***MenSCs and BMSCs improved the renal and hepatic functional profile in the MI model***

**Figure 3:**
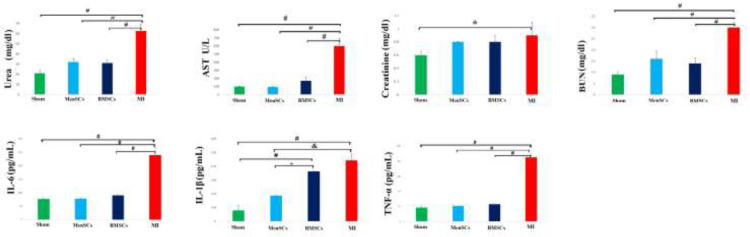
Urea, AST, creatinine, and BUN levels, as markers of hepatic and renal function and Pro-inflammatory cytokines level of IL-6, IL-1β, and TNF-α in plasma following MI in sham, MI, BMSCs, and MenSCs received groups. *P<0.05, &*P*<0.01, and # *P*<0.001

AST: Aspartate aminotransferase; BUN: Blood Urea Nitrogen; IL-6: Iterleukin; TNF-α: Tumor necrosis factor-α; MI: Myocardial infarction; BMSCs: Bone marrow stem cells; MenSCs: Menstrual blood stromal (stem) cells

Biochemical indicators of hepatic and renal function were assessed in serum samples. Serum levels of AST, Urea, BUN, and creatinine were significantly increased after MI compared with the sham group (*P<*0.001*, P<*0.001*, P<*0.001*, *and *P<*0.05*, *respectively). Indeed, stem cell therapy noticeably alleviated the concentration of AST, BUN, and urea, and improved liver and kidney functions, however, there were no significant differences in AST, urea, creatinine, and BUN levels between MenSCs and BMSCs received groups (*P˃*0.05) (Figure 3).

***MenSCs and BMSCs treatment down-regulated the inflammatory responses following MI***

To determine the effect of MenSCs and BMSCs systemic administration on the immune response following MI, serum levels of TNF-α, IL-1β, and IL-6 were measured. The expression levels of TNF-α, IL-6, and IL-1β were increased substantially in the MI group compared with the sham group (*P<*0.001*, P<*0.001*, and P<*0.001*, *respectively*)*. A significant reduction in the TNF-α and IL-6 serum concentration was observed in the MenSCs and BMSCs received groups in comparison with the MI groups. In addition, MenSCs significantly decreased the IL-1β level compared with the BMSCs group (*P<*0.05) (Figure 3). 

***MenSCs treatment improved renal histopathological alterations following MI***

In the sham group, normal structure of tubules and glomeruli was observed. In the MI group, kidney sections showed signs of degenerative changes, vascular congestion, interstitial inflammation, and some sign of necrosis. In the BMSCs received group, necrosis, hemorrhage, degenerative change, cell swelling, and hyperemia were also seen. In the MenSCs received group, the pathological alteration was alleviated noticeably.

**Figure 4:**
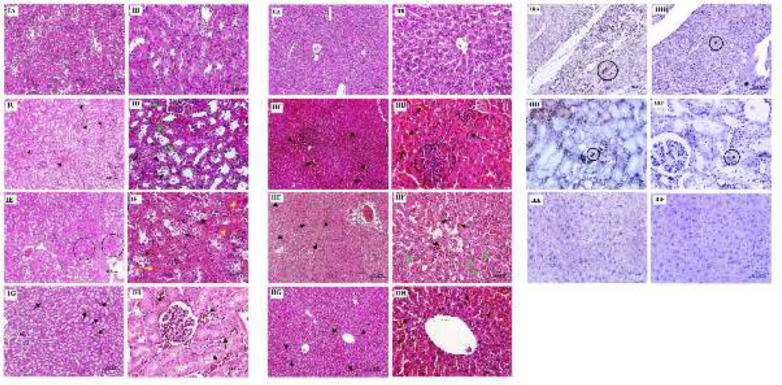
Representative histological images of renal tissue (IA-IH), hepatic tissue (IIA-IIH), and tracking of MenSCs and BMSCs in the cardiac, renal, and hepatic tissues using an anti-human mitochondrial antibody (IIIA-IIIF) 28 days after stem cell administration following MI induction. IA and IB: Sham group with normal morphology, IC: MI group, note vascular congestion (black arrow), ID: Higher magnification of previous figure, note pyknotic nuclei (green arrow) and degenerative changes in tubules (black arrow), IE: BMSCs received group, note foci of coagulative necrosis (circle), IF: Note vacuolar degenerative change (black arrow) and necrotic tubules (yellow arrow) in BMSCs received group, IG: Vascular congestion (black arrow) in MenSCs received group, IH: Previous figure with higher magnification. II A and IIB: Sham group with normal morphology, IIC: MI group, note congestion (black arrow) and focal parenchymal inflammation (circle), IID: Previous figure with higher magnification, IIE: BMSCs received group with congestion (black arrow), IIF: Note sinusoidal dilation (black arrow) and degenerated hepatocytes (green arrow) in BMSCs received group, IIG: MenSCs received group with relatively normal morphology, however congestion (arrow) existed already, IIH: Previous figure with higher magnification; IIIA and IIIB: Tracking of human miothocondiria from MenSCs and BMSCs in the injured heart, IIIC and IIID: Tracking of human miothocondiria from MenSCs and BMSCs in the kidney, IIIE and IIIF: Tracking of human miothocondiria from MenSCs and BMSCs in the liver. Mitochondria were detected only in heart and kidney (circle)

BMSCs: Bone marrow stem cells; MenSCs: Menstrual blood stromal (stem) cells; MI: Myocardial infarction

The liver sections of the sham group were demonstrated to have normal morphological architecture. In the MI group, mild parenchymal inflammation and degenerative changes were the prominent pathological findings. In the BMSCs received group, dilation of sinusoidal space and degeneration with mild intensity were detected. In the MenSCs received group, only congestion was detected as pathologic changes (Figure 4 IA-IIH).

***MenSCs and BMSCs migrated successfully into the injured sites after systemic administration***

Twenty eight days after intravenous injection, homing of MenSCs and BMSCs was tracked in the injured area. IHC staining of heart, kidney, and liver sections using the anti-human mitochondrial antibody showed that systemic injection of MenSCs and BMSCs resulted in homing of these cells into the cardiac and renal tissues. However, none of them were detected in hepatic tissue (Figure 4 IIIA-IIIF).

Successful transfer of human mitochondria from MenSCs in the injured heart (**IIIA**) and kidney (**IIIC**) and also from BMSCs in the injured heart (**IIIB**) and kidney (**IIID**). However, with respect to mild damage in hepatic tissue following MI, no mitochondrial transfer from MenSCs (**IIIE**) and BMSCs (**IIIF**) in hepatic tissues was detected. 

***MenSCs treatment prevented apoptosis following MI evaluated by detection of DNA fragmentation in renal tissue***

**Figure 5:**
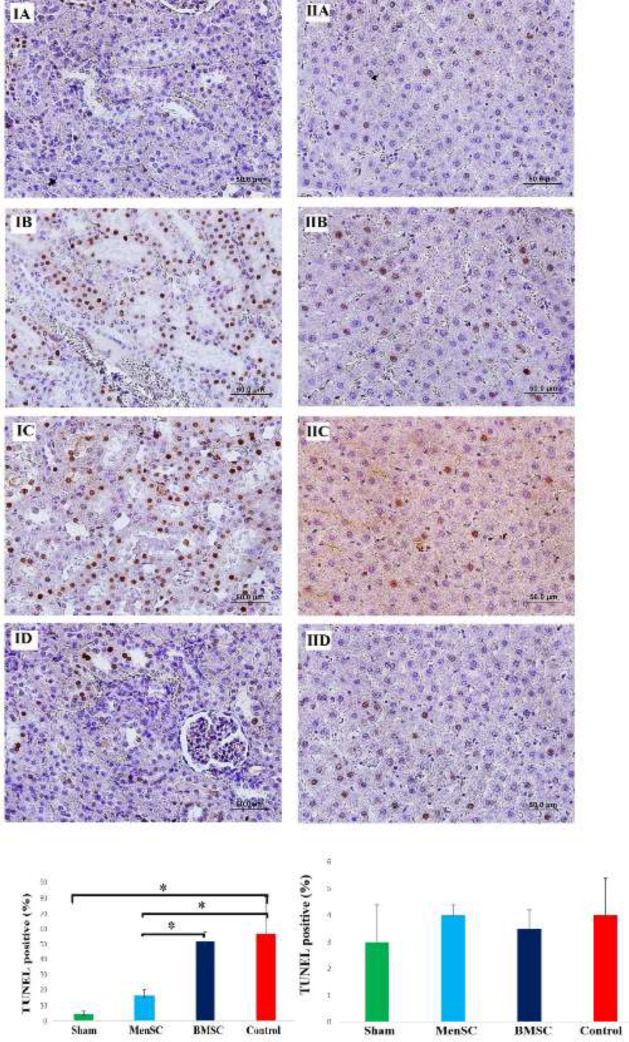
Detection of DNA fragmentation using the TUNEL method in the kidney (IA- ID) and liver (IIA- IID) after MI induction. Brown nuclei indicate apoptotic cells. IA: Sham, IB: MI, IC: BMSCs received, and ID: MenSCs received. Administration of MenSCs contributed to reduced cell apoptosis in the injured kidney. There were no significant differences in cell apoptosis between the BMSCs received group and the MI group. IIA: Sham, IIB: MI, IIC: BMSCs received, and IID: MenSCs received, note that a low number of apoptotic cells were observed in hepatic tissue of all groups with no significant difference. *P<0.05, (Scale bars=50 mm)

BMSCs: Bone marrow stem cells; MenSCs: Menstrual blood stromal (stem) cells; MI: Myocardial infarction

In renal tissues belonging to the sham group, TUNEL-positive cells were observed rarely. The number of TUNEL-positive cells was markedly increased in the MI group in comparison with the MenSCs and sham groups (*P<*0.05 and *P<*0.05*,* respectively). Systemic administration of MenSCs significantly reduced apoptotic cell death compared with the BMSCs group (*P<*0.05). No significant difference was observed in the BMSCs group and MI group (*P˃*0.05). According to TUNEL staining results, a low number of apoptotic cells were observed in the hepatic tissues of all groups. Overall, no significant difference was detected among groups (*P˃*0.05) (Figure 5).

***MenSCs administration decreased the expression of NF-κB levels in renal tissue following MI***

**Figure 6:**
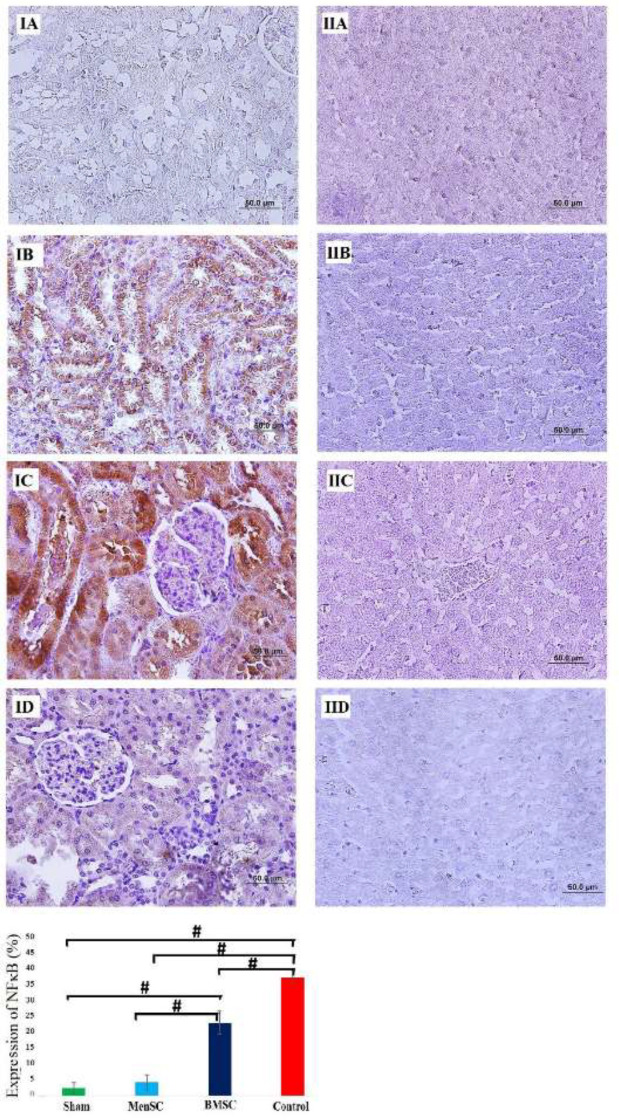
IHC staining for detection of NF-κB expression in renal (IA-ID) and hepatic (IIA-IID) tissues. IA & II A: Sham, IB & IIB: MI, IC & IIC: BMSCs received, and ID & IID: MenSCs received. MenSCs reduced the expression level of NF-κB expression in the kidney. No NF-κB expression was detected in hepatic tissues from any group. Brown color indicates NF-kB positivity, #*P*<0.001, (Scale bars=50 mm)

IHC: Immunohistochemistry; BMSCs: Bone marrow stem cells; MenSCs: Menstrual blood stromal (stem) cells

Activated NF-κB was assessed by immunocytochemistry and showed many positively stained cells in the epithelial cells of renal tubules belonging to the MI group and BMSCs received group. However, administration of MenSCs seven days after MI induction could significantly decrease the NF-κB activity in renal tissue (*P<*0.001). In hepatic tissue, no significant NF-κB expression was observed in any group (Figure 6). 

## Discussion

Insufficient blood supply to the vital organs following MI can lead to permanent and progressive damages (27, 28). Deleterious effects of cardiac ischemia on distant organs have been recognized by various mechanisms such as hemodynamic alterations, ROS production, endothelial dysfunction, and inflammation (6). Several studies have described the beneficial effect of MenSCs administration after MI (29). However, the potency of these cells compared with BMSCs in alleviating the outcomes of MI on distant organs has not been explored. 

We have shown a significant reduction in the level of AST, creatinine, urea, and BUN in both MenSCs and BMSCs received groups compared with the MI group. We also demonstrated that MenSCs possessed more therapeutic potency in comparison with BMSCs and could appropriately protect kidneys from subsequent injuries following MI with decreased cell death (apoptotic and necrotic cell) and modulation of inflammatory reaction. However, in hepatic tissue, no serious irreversible injuries were detected following MI. It is supposed that the damage caused by HF can affect liver function in the long term (30). Indeed, the liver receiving a dual blood supply from the hepatic portal vein and hepatic arteries might play a role in its resistance to hypoxic-induced injuries more than the renal tissue (31). 

We have also demonstrated that pro-inflammatory cytokines increased significantly in the serum of the MI group in reference to both MenSCs and BMSCs administrated groups. Inflammatory cytokines could release from the ischemic myocardium as part of the innate and adaptive immune response, which can directly affect distant organs (32). Several studies have provided evidence that MI can promote progressive renal fibrosis and chronic impairment by elevated levels of pro-inflammatory mediators (33, 34). Ruparelia *et al.* have shown increased circulation of neutrophils and monocyte, and release of local inflammatory mediators in the kidney after MI (35). Another study indicated that TNF-α and IL-6 levels were significantly elevated in patients suffering from cardiorenal type 1 (36). Robust evidence displays that MenSCs and BMSCs have immunosuppressive capacity in inflammatory conditions (37, 38); so, the reduction of circulating cytokines may be a result of the paracrine effect of MenSCs and BMSCs. We also demonstrated that the expression of IL-1β was considerably lower in the MenSCs administrated group compared with the animals that received BMSCs. IL-1β promotes renal stromal cell proliferation, deposition of the fibrotic matrix, and tubulointerstitial fibrosis (39). Inhibition of IL-1β can have protective effects on renal disease progress by reduced mRNA expression of neutrophil gelatinase–associated lipocalin as a kidney injury marker and fibrosis (40). MenSCs can induce immunomodulatory properties by suppressing pro-inflammatory factors such as Interferon-γ (IFN-γ) and TNF-α, and secreting anti-inflammatory cytokines like IL-10 and also by interacting with lymphocytes (41). Furthermore, suppression of IL-1β may be related to the property of MenSCs in modulating macrophages central sources of inflammatory cytokines producer (42). Moreover, we indicated that MenSCs can inhibit NF-κB expression more effectively than BMSCs in injured sites. Impairment in myocardial function occurred after MI and can lead to a decrease in cardiac output. This event exacerbates renal ischemia through a reduction in renal blood flow (43). Accumulation of ROS following hypoxic damage to the kidney can activate the NF-κB expression pathway (44). NF-κB has a key role in the induction and regulation of inflammation in many pathological conditions that can lead to functional and structural abnormalities, and consequently cell death (45). Therefore, inhibition of NF-κB can lead to suppression of cytokine release, macrophage infiltration, accumulation of interstitial inflammatory cells, and apoptosis (46). Kumar *et al.* reported that treatment of the renal injury induced animals with NF-κB inhibitor rendered protection against oxidative stress (47). A study reported that MenSCs could down-regulate ROS levels by transporting miRNA Let-7 into the alveolar epithelial cell (48). Another study exhibited that MenSCs-derived CM could reduce the generation of ROS in human neuroblastoma cells exposed to the neurotoxicant 1-methyl-4-phenylpyridinium (22). Thus, it is reasonable to suggest that the suppression of ROS by MenSCs might lead to the inhibition of NF-κB. In addition, the results of the TUNEL assay showed a significant increase in the number of TUNEL-positive cells in the MI group compared with the MenSCs administrated group and interestingly, MenSCs could significantly decrease TUNEL positive-stained cells more than BMSCs in kidney tissue. In another study, the effect of human amniotic fluid stem cells and adipose tissue stromal vascular fraction cells on kidney function after HF were examined. They showed that both cells can improve kidney function by reducing apoptosis, pro-inflammatory cytokines, and differentiation into tubular, endothelial, and smooth muscle cells (49).

Tissue regeneration following stem cell therapy may depend on homing of stem cells into the injured site. Stromal cell-derived factor-1a (SDF-1) plays a critical role in cell trafficking and directing stem cells to the site of injury (50). During injury, cells from the damaged organ highly express SDF-1, which leads to homing of stem cells to the damaged tissue (51). In this study, we indicated that MenSCs and BMSCs could migrate to the damaged areas of the heart and kidney, but none of them were detected in hepatic tissue. Limited tissue damage and lack of NF-κB expression in liver tissue post-MI may lead to decreased SDF-1 expression and eventually lack of stem cell migration to hepatic tissue.

## Conclusion

To conclude, organ crosstalk during HF has a noticeable impact on remote organ-system comorbidities. Since inflammation plays the main role in remote organ injury following MI, our findings demonstrate that compared to BMSCs, MenSCs can be more effective in protection against remote organ injuries following MI. However, there are some challenges in the use of stem cells to overcome multi-organ dysfunction after MI. For example, is the beneficial therapeutic effect of stem cells on remote organs after MI long-term? Can direct injection of the stem cells into the injured tissue after MI be more effective than their systemic injection? Therefore, more studies are needed to investigate the exact mechanism of stem cell therapy on distant organs after MI.

## Authors’ Contributions

SK, NA, and HG designed the experiments; MM, HG, MD, and NN performed experiments and collected data; MM and HG discussed the results and strategy; SK, NA, and HG supervised, directed, and managed the study; SK, NA, MM, MD, ND, and HG approved the final version to be published.

## Funding Source

The authors would like to thank the authorities of Iran University of Medical Sciences, Tehran, Iran, for their financial support (grant number: 99-2-4-17061) and for providing facilities for performing this research.

## Conflicts of Interest

The authors have no conflicts of interest to declare.
